# Cardiac prostheses‐related hemolytic anemia

**DOI:** 10.1002/clc.23191

**Published:** 2019-05-06

**Authors:** Mohamad Alkhouli, Ali Farooq, Ronald S. Go, Sudarshan Balla, Chalak Berzingi

**Affiliations:** ^1^ Division of Cardiology, Department of Medicine West Virginia University Morgantown West Virginia; ^2^ Division of Cardiology, Department of Medicine West Virginia University Charleston West Virginia; ^3^ Division of Hematology, Department of Medicine Mayo Clinic Rochester Minnesota

**Keywords:** anemia, cardiac prosthesis, hemolysis, left ventricular assist device, paravalvular leak

## Abstract

Hemolysis is an unintended sequel of temporary or permanent intracardiac devices. However, limited data exist on the characteristics and treatment of hemolysis in patients with cardiac prostheses. This entity, albeit uncommon, often poses significant diagnostic and management challenges to the clinical cardiologist. In this article, we aim to provide a contemporary overview of the incidence, mechanisms, diagnosis, and management of cardiac prosthesis‐related hemolysis.

## 1 INTRODUCTION

Cardiac prosthesis‐related hemolytic anemia (CPHA) is a well described but likely an under‐recognized phenomenon. This potentially life‐threatening complication was first described in the 1950s to 1960s in patients undergoing valve replacement with early generation surgical prostheses.^1^, ^2^ The incidence of clinically evident hemolysis after surgical valve replacement has since declined due to the improved valve design and surgical implantation techniques.^3^, ^4^ However, interest in CPHA has been recently renewed given the increasing number of studies reporting various rates of clinical and subclinical CPHA with mechanical circulatory support devices and transcatheter valvular interventions.^5^, ^6^, ^7^ Nonetheless, the management of CPHA is often challenging due to its atypical presentation, lack of standardized definitions/classifications, and due to the dearth of outcomes data on its various treatment strategies. We sought to provide a contemporary overview of the current literature on the incidence, mechanisms, and management strategies of hemolytic anemia associated with various cardiac prostheses.

## 2 DEFINITION OF HEMOLYTIC ANEMIA

There is no single specific definition of hemolytic anemia. However, the diagnosis of hemolytic anemia is usually established if three major criteria are present: (a) unexplained anemia, and (b) signs of accelerated right blood cells (RBCs) production in the bone marrow (eg, high reticulocyte count), and (c) signs of RBCs destruction (eg, elevated unconjugated bilirubin, lactate dehydrogenase [LDH], low haptoglobin). The term “sub‐clinical hemolysis” is used to describe patients who meet the latter two criteria but do not have anemia. In these patients, the bone marrow adequately compensate for the hemolysis, maintaining normal hemoglobin. Prosthesis‐related hemolytic anemia can then be assumed if new hemolysis is diagnosed in patients with cardiac prostheses, and/or mechanical assist devices in the absence of other causes of hemolysis.

## 3 INCIDENCE AND ETIOLOGY OF CARDIAC PROSTHESIS‐RELATED HEMOLYSIS

The incidence of hemolysis in patients with cardiac prostheses varies widely according to the device type and its indwelling time. Mechanical damage to the RBCs due to increased shear stress is the most widely accepted etiology of CPHA. However, causes of this increased shear stress are device‐ and disease‐specific.

### 3.1 Hemolysis after open valve surgery

Hemolytic anemia was a common complication of old generation valves, occurring in up to 15% of surgical valves in the 1960s to 1970s.^8^, ^9^ However, this incidence decreased to <1% with modern valve designs. In a study of 301 patients who underwent On‐X mechanical valve replacement, clinical hemolysis at long‐term occurred in 0% and 0.2% of patients who had aortic and mitral valve replacement, respectively.^3^ Several other studies confirmed the rarity of clinical hemolysis after valve replacement with contemporary prostheses.^4^, ^10^, ^11^ Nonetheless, subclinical hemolysis is not uncommon, occurring in 18% to 51% and in 5% to 10% of contemporary mechanical tissue prostheses, respectively.^12^, ^13^ The main mechanism of hemolysis after surgical valve replacement is paravalvular leak (PVL), which may result from suture dehiscence due to heavy annular calcifications, endocarditis, chronic steroids, or suboptimal surgical techniques.^14^, ^15^, ^16^, ^17^ Other less common etiologies of hemolysis related to surgical prostheses are listed in Table 1.^18^, ^19^, ^20^, ^21^

**Table 1:** Mechanisms of cardiac prosthesis‐related hemolysis

Cardiac device	Mechanisms of hemolysis
Surgical aortic and mitral valve replacement	PVL, SVD, PPM, endocarditis, leaflet thrombosis
Surgical mitral valve repair	Ring dehiscence, residual eccentric or para‐ring regurgitation, protrusion of suture material, free‐floating chordae in hyperdynamic left ventricle
Transcatheter aortic valve replacement	PVL, PPM, increased red cell shear stress in the sinuses due to residual native valve fissuring and balloon‐induced endothelial denudation
Transcatheter mitral valve replacement	PVL
Surgical left ventricular assist devices	Pump thrombosis, transfusion‐associated hemolysis, cannula kinks or malposition, dehydration → LV under filling → increased inlet velocity
Percutaneous left ventricular assist devices	Pump‐related shear stress, device malpositioning, device malfunction
Intracardiac shunt closure	Incomplete closure (peri‐device leak)

Abbreviations: LV; left ventricle; PVL, paravalvular leak; PPM; patient‐prosthesis mismatch; SVD, structural heart deterioration.

Hemolysis also complicates a small percentage (<1%) of mitral valve repair and annular ring placement surgeries.^22^, ^23^, ^24^, ^25^ Although ring dehiscence appears to be the main mechanism of CPAH in this group, other reported mechanisms include: protruding of the paravalvular suture material, “whiplash motion” of residual free‐floating chordae in hyperkinetic ventricles, and small but turbulent eccentric residual regurgitation jet (Table 1). In this large series, valve replacement led to the resolution of hemolysis in the vast majority of cases.^26^, ^27^

### 3.2 Hemolysis after transcatheter valve replacement

**Figure 1:**
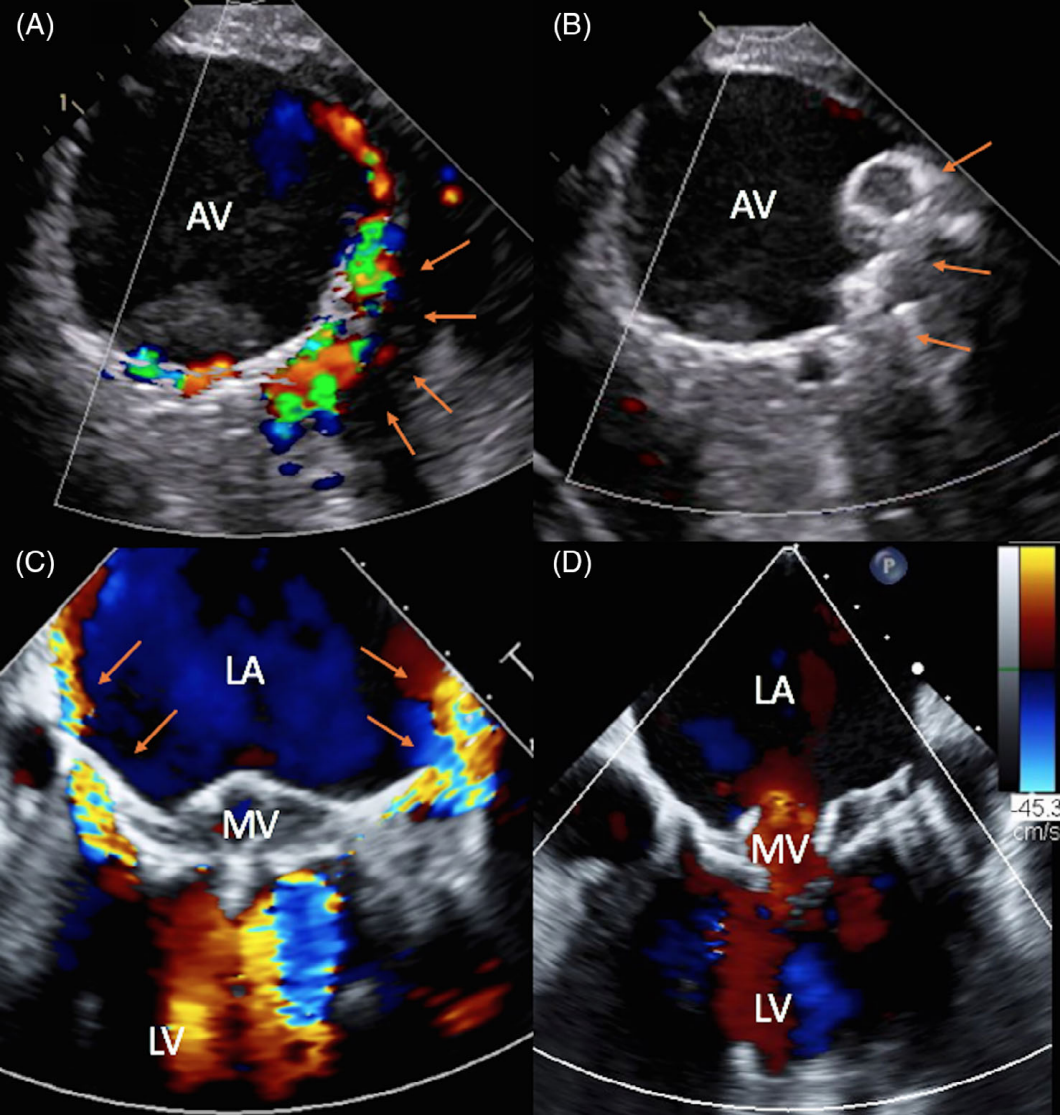
Severe hemolysis due to paravalvular leak after transcatheter aortic and mitral valve replacement. A,B, Paravalvular leak after transcatheter aortic valve replacement before and after percutaneous closure. C,D, Paravalvular leak after TMVR before and after a second procedure to reposition the mitral prosthesis. AV, aortic valve; LA, left atrium; LV, left ventricle; MV, mitral valve; arrows annotate the location of the paravalvular leak

The incidence of hemolysis after transcatheter aortic valve replacement (TAVR) is unknown because routine surveys are not performed in these patients. Although clinical hemolysis is not commonly seen, subclinical hemolysis following TAVR may not be uncommon.^17^, ^28^ In a study of 122 patients who had TAVR with balloon‐expandable valves, subclinical hemolysis occurred in 15%. The strongest predictor of hemolysis was patient‐prosthesis mismatch, rather than the degree of PVL.^7^ This intriguing finding, albeit requires confirmation in additional studies, suggests that transcatheter (vs surgical) prostheses may be associated with less hemolysis given their lower reported incidence of patient‐prosthesis mismatch.^29^ In another study of 64 TAVR patients, 37.5% had evidence of subclinical hemolysis 6 months after the procedure.^30^ Moderate to severe PVL and bicuspid valve morphology independently predicted hemolysis (Figure 1A,B), and hemolysis was associated with a 4‐fold increase in hospital readmissions at 1 year. Of note, 21% of patients had evidence of sub‐clinical hemolysis before TAVR; supporting the notion that severe native aortic stenosis can lead to hemolysis due to flow acceleration across the stenotic valve.^31^, ^32^ Other TAVR‐specific mechanisms of CPAH are related to the remaining native leaflets and their potential impact on red cell shear stress (Table 1).^7^

The field of transcatheter mitral valve replacement (TMVR) is rapidly evolving.^33^ However, given the small number of TMVRs performed worldwide, data on TMVR associated hemolysis are limited. In the early feasibly trial of the Tendyne valve (Abbott, Roseville, Minnesota), only 1 of 30 patient (3.3%) developed severe hemolysis.^34^ The main mechanism of hemolysis after TMVR is PVL due to incomplete sealing, device undersizing, or progressive left ventricular remodeling (Figure 1C,D). Cases of severe clinical hemolysis have also been reported following transcatheter mitral valve in valve/ring implantation.^35^

### 3.3 Hemolysis with left ventricular assist devices

The reported incidence of hemolysis with the HeartMate II (HMII; Thoratec, Pleasanton, California) is approximately 13% to 18%.^5^, ^36^, ^37^ However, early experience with the third generation magnetically levitated left ventricular assist devices (LVAD) (HeartMate III) revealed very low (<1%) rates of hemolysis.^38^, ^39^ Similarly, the novel TORVAD toroidal‐flow LVAD has shown negligible rates of hemolysis in pre‐clinical testing.^40^ A unique aspect of hemolysis in LVAD patients is its strong relationship with thrombotic complications. Local thrombosis increases shear stress and leads to local destruction of the red cells.^41^ However, other mechanisms may be implicated such as increased inlet velocities due to dehydration and under‐filling of the left ventricle, transfusion‐associated hemolysis, and cannula kinks or malposition (Table 1).^41^, ^42^, ^43^ In current practice, an increase in LDH and plasma free hemoglobin levels in LVAD patients is viewed as a possible early cannula thrombosis.^5^ Clinical hemolysis in surgical LVAD patients is associated with significant morbidity and mortality.^44^, ^45^, ^46^

Mild hemolysis occurs in 10% to 30% of patients who receive short‐term percutaneous LVAD support with the Impella device (Abiomed Inc., Danvers, Massachusetts).^47^, ^48^, ^49^ However, the incidence increases to 60% with device indwelling times >6 hours.^6^ In a study of patients undergoing veno‐arterial extracorporeal membrane oxygenation, concomitant Impella use was associated with higher incidence of hemolysis (76% vs 33%, *P* = .004).^50^ Clinically significant hemolysis may also occur unexpectedly due to device malfunction or improper placement.^51^, ^52^, ^53^ To the best of our knowledge, no cases of clinical hemolysis due to intra‐aortic balloon pumps have been reported.

### 3.4 Hemolysis after transcatheter shunt closure

**Figure 2:**
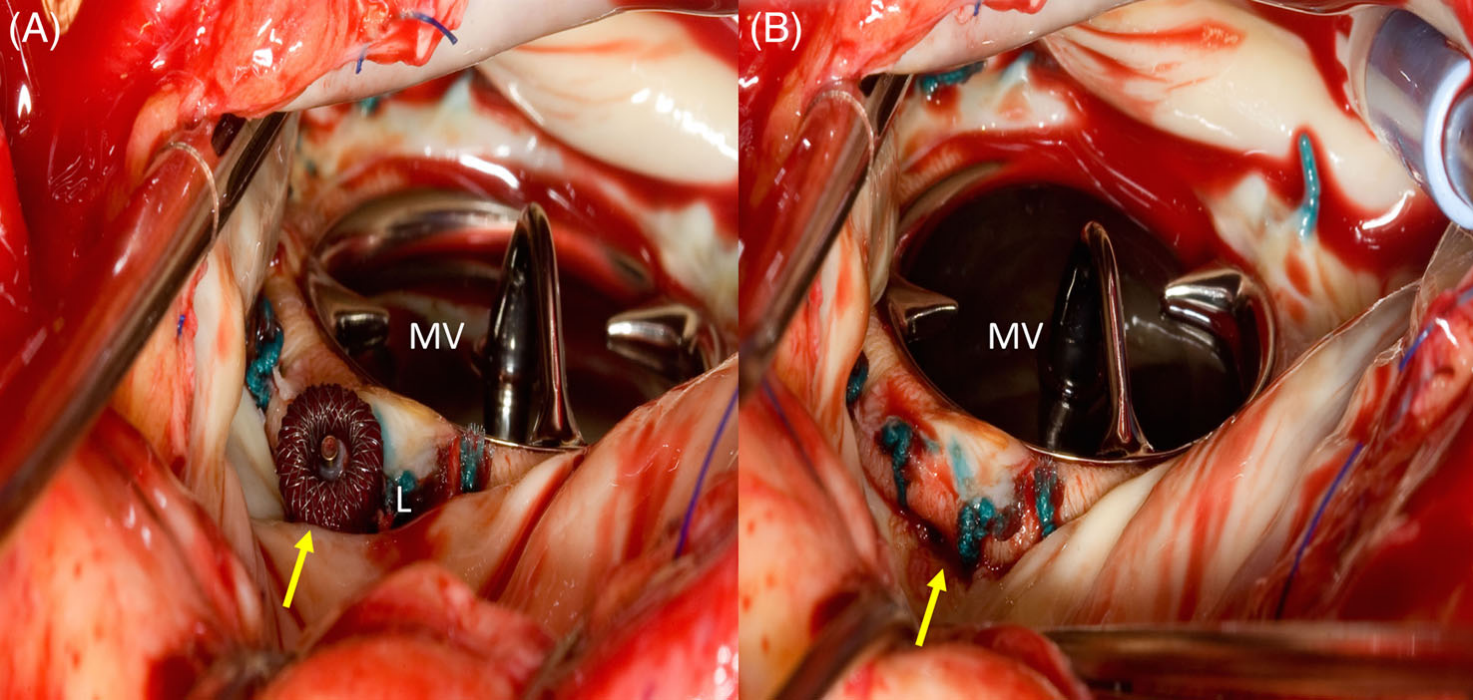
Redo surgery for severe hemolysis following failed percutaneous paravalvular leak closure attempt. A, Amplatzer vascular plug in place, but residual leak is present (L). B, Removal of the Amplatzer vascular plug followed by patch repair of the leak. MV, mitral valve, arrows annotate the location of the paravalvular leak

New hemolysis requiring blood transfusion occurred in 1% to 2% of patients undergoing percutaneous PVL closure in two large registries in the United Kingdom and the United States, likely due to incomplete obliteration of the PVL (Figure 2).^28^, ^54^ Higher profile devices (eg, ventricular septal occluders) are more associated with more hemolysis than the lower profile Amplatzer vascular plugs.^55^, ^56^ Severe hemolysis has also been reported following percutaneous closure of septal defects and peri‐MitraClip regurgitation mostly due to residual peri‐device shunt.^57^, ^58^, ^59^, ^60^, ^61^, ^62^, ^63^

## 4 CLINICAL PRESENTATION AND DIAGNOSTIC APPROACHES

**Figure 3:**
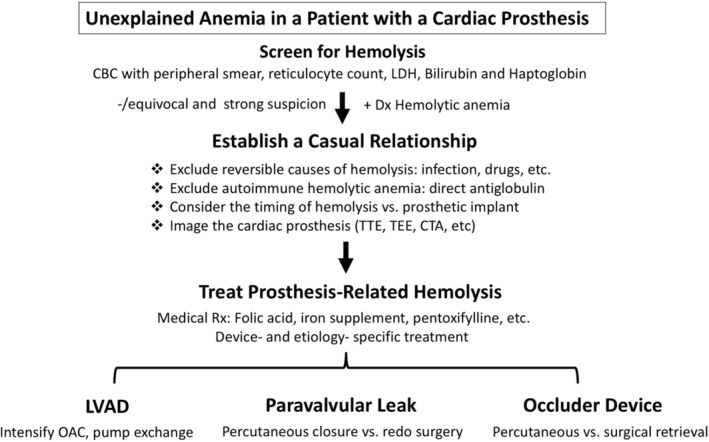
An algorithmic approach to a patient with suspected cardiac prosthesis‐related hemolysis. CTA; computed tomography angiogram; LDH, lactate dehydrogenase; LVAD, left ventricular assist device; OAC, oral anticoagulation; TEE, transesophageal echocardiography; TTE, transthoracic echocardiography

The recognition of hemolytic anemia in patients with classic presentations is straightforward. However, CPHA commonly presents with ambiguous symptoms, and insidious onset posing highlighting the need for a high index of suspicion. In a study of 381 patients who were referred for treatment of mitral PVL (of whom 40% had hemolysis), the mean time from index valve replacement to referral was 85.1 ± 115.6 months.^64^ Hence, in patients with cardiac prostheses who have unexplained anemia, a systematic step‐wise approach to exclude/diagnose CPHA is warranted (Figure 3):

### 4.1 Laboratory confirmation of hemolysis

Initial testing should include: complete blood count with peripheral blood smear examination, reticulocyte count, LDH, bilirubin, and haptoglobin levels. The normal values, accuracy, and pitfalls of these tests are summarized in Table 1.

(a) Blood smear examination: erythrocyte fragments (eg, Schistocytes) are common in “mechanical” hemolytic anemia. These fragments, however, are not specific to CPHA (Table 1). (b) Reticulocytes: an elevated reticulocyte count is a typical feature of hemolysis. However, reticulocytes can be elevated in other causes of accelerated red cells production (Table 1). In addition, normal or low reticulocyte counts do not preclude hemolysis. A blunted bone marrow response can be seen with myelodysplasia, alcoholism, or iron and folate deficiency. (c) LDH: this enzyme catalyzes the conversion of lactate into pyruvic acid, and its iso‐enzymes LDH‐1 and 2 are therefore increased in hemolysis. Although non‐specific, an elevated LDH >2.5‐folds strongly suggests hemolysis. Temporal trends in LDH are also useful in assessing treatment success in patients with PVL and LVAD dysfunction. (d) Haptoglobin: this scavenger binds free circulating hemoglobin released with RBC turnover, and it hence becomes diminished or undetectable in significant hemolysis. A haptoglobin level < 25 mg/dL provides an 87% probability of having hemolysis.^65^ A combination of haptoglobin <25 mg/dL and elevated LDH increases the predictive value to >90%. However, haptoglobin is an acute phase reactant and its level can hence be normal or elevated in systemic inflammation or acute infection. (e) Bilirubin: indirect bilirubin is a product of hemoglobin catabolism, and its levels are thus increased in hemolysis. Although severely elevated unconjugated bilirubin can occur with acute massive hemolysis, levels >4 mg/dL in non‐acute hemolysis typically indicate a concomitant liver pathology impairing the conjugation of bilirubin or its hepatic uptake.

Additional markers of hemolysis can be used in certain populations. For example, plasma free hemoglobin (pFH) is usually indicative of intra‐vascular hemolysis. This marker is useful in early detection of hemolysis in patients with LVADs. In the Interagency for Mechanically Assisted Circulatory Support registry, hemolysis is diagnosed when pFH exceeds 40 mg/dL.^46^ The utility of pFH in diagnosing and monitoring hemolysis in patients with pLVAD and in those with PVL has also been suggested in several studies.^49^, ^66^ Indeed, pFH was superior to LDH in detecting hemolysis in patients with cardiogenic shock treated with the Impella micro‐axial pump.^49^ However, pFH is not widely used in this setting due to the dearth of supportive data and the frequent need to send out to a reference lab. Other tests indirect/less specific markers of hemolysis (mean cell volume, hemosiderinuria, hemoglobin A1C, aspartate aminotransferase, etc.) may aid in establishing or excluding the diagnosis in equivocal cases (Table 2).

**Table 2:** Markers of hemolysis

Type	Test	Findings in hemolysis	Normal values	Characteristics/pitfalls
Direct	Haptoglobin	<25 mg/dL	0.5‐3.2 g/L	Most specific
				↑ = Acute phase reactant, nephrotic syndrome, wide range of normal values
				↓Trauma, congenital ahaptoglobinemia, cirrhosis
	Lactate dehydrogenase	>460 μ/L	230‐460 μ/L	Non‐specific but LDH + ↓ Haptoglobin>90% specific for hemolysis
				Other sources of LDH increase liver, myocardial, or muscle injury
	Indirect bilirubin	>2 mg/dL	0.3‐1.6 mg/dL	Non‐specific
	Aspartate aminotransferase	>40 μ/L	10‐40 μ/L	Non‐specific
	Cell deformities on peripheral smear, “eg, schistocytes”	>0.5%	Absent	Non‐specific, also seen in DIC, thrombotic microangiopathies, spherocytes, elliptocytes, and sickle cells can be seen
Indirect	Reticulocyte count	>2%	<2%	↑ With bleeding and erythropoietin use
				↓ with myelodysplasia, alcohol, B12/folate/iron deficiency
	Mean cell volume	>96 femtoliters	80‐96 femtoliters	↑ Due to reticulocytosis
	Hemosiderinuria	Brown‐color urine	Absent	More characteristics of acute and marked hemolysis, which is uncommon with cardiac etiologies of hemolysis
	Hemoglobin A1C	Unexpectedly low	≤5.6	Limited time for red blood cell glycation
	Plasma free Hgb	>40 mg/dL	<5 mg/dL	Not widely available (reference labs)

Abbreviations: Hgb, hemoglobin; DIC, disseminated intravascular coagulation; LDH, lactate dehydrogenase.

### 4.2 Establishing the relationship between cardiac prostheses and hemolysis

Once the hemolysis diagnosis is confirmed, establishing its relationship with cardiac prostheses is essential to guide therapy. Although this can be challenging due to the absence of a specific test for CPHA, the following steps may be helpful in elucidating the etiology of hemolysis.

1. Excluding common causes of hemolysis: infection and drugs are frequent causes of hemolysis. Discontinuation of the potential offenders (eg, new antibiotics), and treating underlying infections may resolve the hemolysis. Autoimmune hemolytic anemia should also always be excluded with a direct antiglobulin test before confirming the diagnosis of CPHA.

2. Identifying specific laboratory clues: certain laboratory findings may suggest a specific etiology for the hemolysis. For example, the presence of spherocytes and/or a positive direct antiglobulin test suggest an immune‐mediated mechanism, while the abundance of schistocytes indicates mechanical disruption of the red cells. Hence, the latter pattern is more likely to be observed in patients with CPHA, although it can also be seen with thrombotic thrombocytopenic purpura or hemolytic uremic syndrome.

3. Imaging the cardiac prosthesis: echocardiography allows detailed evaluation of prosthetic valve (transvalvular velocity, leaflet function, PVL, etc.), LVADs (position and integrity/kinks of the cannula/pump, function of aortic valve, etc.), and intra‐cardiac shunt occluder devices (eg, residual leak). Although transthoracic echocardiography can often identify the site and mechanism of prosthesis dysfunction, transesophageal echo is usually necessary. Cardiac computed tomography might also be useful in confirming the diagnosis and guiding treatment of PVL, peri‐occluder residual shunting, and LVAD outflow graft kinks.^17^, ^42^

4. Timing of presentation: Severe anemia after LVAD insertion or valve replacement should raise suspicion of a causal relationship between the cardiac prosthesis and hemolysis in the absence of bleeding or infection. Nonetheless, CPHA can often be insidious and may not be detected clinically until later stages.^64^

## 5 MANAGEMENT OF CARDIAC PROSTHESIS‐RELATED HEMOLYSIS

The optimal treatment strategy of cardiac prosthesis‐related hemolysis is determined by the degree of hemolysis, clinical symptoms, severity of prosthetic dysfunction, and the predicted risk and success of surgical or percutaneous interventions.

### 5.1 Medical therapy

Medical therapy with close follow‐up is appropriate for patients with mild hemolysis that is not significantly interfering with the quality of life.

- Folic acid: Folate deficiency is common in chronic hemolysis due to the increased consumption from accelerated erythropoiesis.^67^ In persistent hemolysis, prophylactic oral folic acid supplementation is recommended to avoid substantial folate deficiency.
- Iron supplementation and blood transfusion: Oral ± intravenous iron supplements may be sufficient to treat stable degrees of hemolysis. However, blood transfusion is often needed is severe hemolysis until mechanical corrective measures are undertaken.
- Beta‐blocker: Beta‐blockers can reduce shear forces in patients with PVL‐related hemolysis reducing blood pressure and heart rate. Oral beta‐blockers led to significant improvement in hemolytic anemia in several retrospective series.^68^, ^69^, ^70^
- Pentoxifylline: Pentoxifylline improves blood viscosity and erythrocyte deformability. The use of pentoxifylline may subside mild hemolysis in patients with LVAD or mechanical valves.^71^, ^72^ In a small randomized trial of 40 patients with CPHA, hemolysis indices improved in 60% of patients on pentoxifylline compared to 5% of patients in the placebo group.^73^
- Erythropoietin: This recombinant hormone has been shown to eliminate the need for transfusion in selected patients with prosthetic valve‐related hemolysis.^74^, ^75^ However, erythropoietin administration to treat LVAD‐related hemolysis was associated with higher odds of pump thrombosis (hazard ratio 2.35; 95% confidence interval: 1.38‐4.00; *P* = .002), and mortality (hazard ratio 1.62; 95% confidence interval: 1.12‐2.33; *P* = .01).^76^
- Anticoagulation: Intensification of antithrombotic therapy is the first recommended step in the management of LVAD associated hemolysis that is believed to be due to thrombosis.^41^, ^77^

### 5.2 Invasive management

Invasive treatment is reserved for patients with severe symptomatic hemolysis despite maximal medical therapy. The specific invasive treatment differs according to the implicated cardiac prosthesis and the underlying mechanism of hemolysis.

Paravalvular leak repair: The efficacy of transcatheter PVL repair in reducing heart failure symptoms and long‐term mortality has been demonstrated in multiple studies.^28^, ^64^, ^78^ However, the literature on the role of transcatheter PVL correction in resolving hemolysis is scarce, and conflicting. For example, Ruiz et al reported a substantial decrease in the percentage of patients who required blood transfusion or erythropoietin injections after percutaneous PVL repair from 56% to 5%.^55^ On the contrary, in another study of 168 patients with PVL, blood transfusion requirements decreased only modestly after percutaneous repair from 34% to 21%.^79^ These inconsistencies may be related to the variable definitions of hemolysis and the degree of PVL reduction achieved. Several studies have demonstrated that effective correction of hemolysis in these patients requires complete amelioration of the PVL.^16^, ^17^, ^55^, ^64^, ^66^, ^80^ Nonetheless, this can be challenging due to various patients' and device‐specific reasons. Indeed, up to 30% of patients undergoing percutaneous PVL repair have >mild residual PVL following intervention even at centers of excellent.^17^, ^28^, ^64^, ^80^ Among patients with significant residual leaks, a small percentage will experience worsening of hemolysis often requiring percutaneous or surgical device retrieval.^81^ Surgical PVL correction has been shown to be a more effective method in treating severe hemolysis than percutaneous repair.^82^ In one study, persistence of or worsening hemolysis was responsible for 50% of crossovers to surgery in patients initially treated with transcatheter techniques.^64^ However, redo surgery is associated with significant morbidity and mortality, and the choice of transcatheter or surgical intervention requires a collaborative interdisciplinary approach weighting the risks and potential success of each procedure.^78^

Refractory LVAD‐related hemolysis: Intensification of antithrombotic therapy is able to improve or resolve hemolysis in the majority of LVAD patients. However, persistent hemolysis despite maximally tolerated anticoagulation is associated with a substantial increase in the risk of stroke and death.^5^, ^77^ Hence, pump exchange through various surgical techniques (subxiphoid ± thoracotomy or redo sternotomy) should be considered early in these patients.^41^

Occluder devices‐induced hemolysis: Severe hemolysis that developed or worsened after transcathter shunt closure is often due to the residual peri‐device shunt. Those residual shunts can be often ameliorated with additional occluder devices, and/or intra‐device coil deployment within the Nitinol cage of the occluder.^79^, ^83^ However, replacement of the involved prosthesis with a different device or conversion to surgical repair is often required.

## 6 SUMMARY

CPHA is an uncommon but important source of morbidity and mortality in patients undergoing valve surgery, transcatheter structural heart interventions, and mechanical circulatory support device implantations. Knowledge of the incidence, etiologies, and the various treatment strategies is key for effective management of this rare but potentially life‐threatening entity.

## CONFLICT OF INTEREST

The authors declare no potential conflict of interests.
